# Diffusive coupling can discriminate between similar reaction mechanisms in an allosteric enzyme system

**DOI:** 10.1186/1752-0509-4-165

**Published:** 2010-11-30

**Authors:** Ronny Straube, Ernesto M Nicola

**Affiliations:** 1 Systems Biology Group, Max Planck Institute for Dynamics of Complex Technical Systems, Sandtorstraße 1, 39106 Magdeburg, Germany; 2 IFISC, Institute for Cross-Disciplinary Physics and Complex Systems (CSIC-UIB),Campus UIB, E-07122 Palma de Mallorca, Spain

## Abstract

**Background:**

A central question for the understanding of biological reaction networks is how a particular dynamic behavior, such as bistability or oscillations, is realized at the molecular level. So far this question has been mainly addressed in well-mixed reaction systems which are conveniently described by ordinary differential equations. However, much less is known about how molecular details of a reaction mechanism can affect the dynamics in diffusively coupled systems because the resulting partial differential equations are much more difficult to analyze.

**Results:**

Motivated by recent experiments we compare two closely related mechanisms for the product activation of allosteric enzymes with respect to their ability to induce different types of reaction-diffusion waves and stationary Turing patterns. The analysis is facilitated by mapping each model to an associated complex Ginzburg-Landau equation. We show that a sequential activation mechanism, as implemented in the model of Monod, Wyman and Changeux (MWC), can generate inward rotating spiral waves which were recently observed as glycolytic activity waves in yeast extracts. In contrast, in the limiting case of a simple Hill activation, the formation of inward propagating waves is suppressed by a Turing instability. The occurrence of this unusual wave dynamics is not related to the magnitude of the enzyme cooperativity (as it is true for the occurrence of oscillations), but to the sensitivity with respect to changes of the activator concentration. Also, the MWC mechanism generates wave patterns that are more stable against long wave length perturbations.

**Conclusions:**

This analysis demonstrates that amplitude equations, which describe the spatio-temporal dynamics near an instability, represent a valuable tool to investigate the molecular effects of reaction mechanisms on pattern formation in spatially extended systems. Using this approach we have shown that the occurrence of inward rotating spiral waves in glycolysis can be explained in terms of an MWC, but not with a Hill mechanism for the activation of the allosteric enzyme phosphofructokinase. Our results also highlight the importance of enzyme oligomerization for a possible experimental generation of Turing patterns in biological systems.

## Background

The modular structure of biochemical reaction networks greatly facilitates the systematic investigation of their design principles [1,2]. In this way it is often possible to identify small functional units called network motifs [3] which convey a particular functionality. A thorough understanding of the relationship between network design and functionality is not only important for a smart modification and regulation of existing networks, but it is also essential to design novel circuits with prescribed functionality [4,5].

Regulatory properties of cellular networks arise from an interplay between positive and/or negative feedback reactions. These feedback reactions can be effective at the transcriptional level, at the posttranslational level or through allosteric interactions. For example, in transcriptional networks feed forward loops can act as a low pass filter [6] or as a fold-change detector [7] depending on the sign of the genetic interactions. Signal transduction cascades often utilize post-translational modifications such as phosphorylation/dephosphorylation cycles to generate ultrasensitivity [8] or bistability [9]. This behavior is advantageous for cell fate decisions where irreversible switch-like transitions are required, e.g. during maturation [10] or cell-cycle progression [11]. Metabolic enzymes are often regulated through allosteric interactions with positive and/or negative effector molecules. A classical example is the allosteric product activation of the glycolytic enzyme phospho-fructokinase (PFK) which may lead to an oscillatory behavior of the glycolytic pathway [12,13].

So far, the relation between particular molecular reaction mechanisms and the resulting macroscopes behavior has been mainly investigated in well-mixed reaction systems where the dynamics is conveniently described by ordinary differential equations [14,15]. However, if the enzymes in reversible modification cycles are located in different cellular compartments diffusive coupling between neighboring enzyme/substrate molecules may generate steep gradients [16] resulting in front-like wave propagation of phosphoproteins [17]. In that way spatially distributed signaling pathways may create step-like activation profiles that can affect the downstream response of the system in a threshold based manner [18]. Hence, spatial coupling can significantly alter the macroscopic behavior of biochemical reaction systems [19] and bring about new functionality to network motifs [20,21].

In reaction-diffusion systems spiral shaped concentration waves and stationary Turing patterns are among the most fascinating spatiotemporal structures. While spiral waves can occur in systems with excitable and oscillatory reaction dynamics [22,23] Turing patterns typically emerge in activator-inhibitor systems with long range inhibition [24,25]. Here, we investigate the effect of different mechanisms of product activation on the generation of such reaction-diffusion patterns in an enzymatic reaction system centered around the PFK which is a central part of the glycolytic pathway. Under well-stirred conditions this system exhibits oscillatory behavior in both cell free extracts [26,27] and in living cells [28], and diffusive coupling was shown to generate waves of glycolytic activity in yeast extracts [29-31]. Recently, we have observed a novel type of spiral wave behavior in that system [32]. By increasing the overall protein concentration of the extract a transition from outward to inward rotating spiral waves (also known as anti-spirals) was induced. While outward propagating waves have been observed in several biological systems [29,33,34] inward rotating spiral waves were, so far, only observed in purely chemical systems [35,36].

Although we could reproduce the inward propagating waves in numerical simulations with the Goldbeter model [32] the underlying molecular mechanism for their generation is still unclear. The simulations have shown that the negative feedback on the PFK activity, as provided by its substrate ATP, is not required to generate anti-waves. Therefore, we focus here on the allosteric activation of the PFK by its product ADP. Specifically, we address the question whether the symmetry model of Monod, Wyman and Changeux (MWC) [37], as employed in the Goldbeter model, is necessary to generate inward propagating waves or whether a more simple Hill kinetics, as it was used by Sel'kov [12] to model the PFK activation, is suffcient. Since the regulatory properties of the PFK play a key role for the emergence of oscillatory behavior in glycolysis [27,38], in particular in yeast extracts [39], these simple models have been remarkably successful in describing general aspects of glycolytic oscillations [12,40].

The spatio-temporal dynamics of the two PFK effectors ADP and ATP is described by a reaction-diffusion system of the type

(1) $$\partial_{t}u=D\nabla_{x}^{2}u+f(u,p),\;u\in {\mathbb{R}}^{2},\;p\in {\mathbb{R}}^{k},$$

where *D *is a (diagonal) diffusion matrix and *p *denotes the kinetic parameters. The reaction mechanism is encoded in the form of the function *f*(*u*, *p*). Due to the diffusive coupling, the analysis of wave patterns in Eq. 1 is more complicated than the investigation of reaction mechanisms under well-mixed conditions. However, near an oscillatory instability due to a supercritical Hopf bifurcation the spatiotemporal dynamics of Eq. 1 is well described by the complex Ginzburg Landau equation (CGLE) [41]

(2) $$\partial_{t}A=(1+ic_{1})\nabla_{\text{x}}^{2}A+A-(1+ic_{3})|A{|}^{2}A,$$

whose solution types are well known [42]. Here, the complex amplitude *A*(*x*, *t*) describes slow spatio-temporal modulations around the unstable steady state. At the level of the CGLE the details of the molecular reaction mechanism are encoded in the dependence of the two real parameters *c*_1 _= *c*_1_(*D, p*) and *c*_3 _= *c*_3_(*p*) on the original system parameters *p *and *D *in Eq. 1. To find the mapping between the two sets of parameters is tedious, but straight-forward [41] (see Methods). The transition between inward and outward propagating waves is marked by the curve *c*_1 _- *c*_3 _= 0 [43-45] where the region *c*_1 _- *c*_3 _*>*0 corresponds to inward propagating waves in Eq. 1.

By explicitly calculating the two CGLE coefficients *c*_1 _and *c*_3 _we show that inward propagating waves can arise in the Goldbeter model due to the sequential binding of product molecules to the allosteric enzyme as implied in the MWC mechanism. In contrast, in the limit of an in finitely large binding a finity, as implicitly assumed in the Sel'kov model, the formation of inward propagating waves is sup-pressed by a Turing instability. We also find a relation between enzyme cooperativity and the occurrence of inward propagating waves. However, it is not the absolute magnitude of the cooperativity which is important here (as it is for the occurrence of oscillations [40]), but the sensitivity of the co-operativity with respect to changes in the activator concentration. Finally, we observe that the sequential activation mechanism has a stabilizing effect on the wave dynamics. Together, this shows that in the presence of diffusive coupling the particular choice of a molecular mechanism can have a significant impact on the type and the stability of spatio-temporal patterns even though the dynamics under well-mixed conditions is qualitatively the same.

### Model Definitions

#### Sel'kov Model

**Figure 1:**
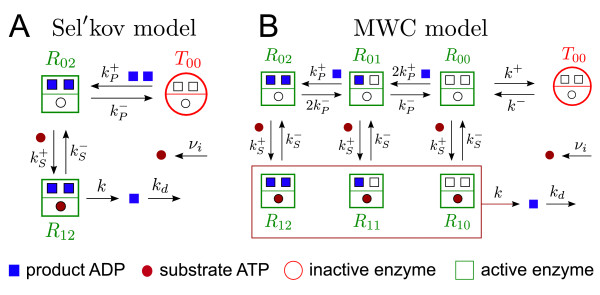
**Reaction mechanisms describing the allosteric activation of the phosphofructokinase by its product ADP**. Hill-like activation as in the Sel'kov model [12] (A) and sequential activation as in the Monod-Wyman-Changeux mechanism [37] used in the Goldbeter model [13] (B) - shown for two ADP-binding subunits (*n *= 2).

In the Sel'kov model it is assumed that the PFK is an oligomeric enzyme which has *n *independent binding sites for the product ADP (activator), but only one binding site for the substrate ATP (inhibitor) [12]. Simultaneous binding of *n *product molecules activates the enzyme which allows for subsequent substrate binding and conversion into product with specific rate *k*. Hence, the Sel'kov model distinguishes only three enzyme states: an inactive state (*T*_00_) which has neither substrate nor product molecules bound and two fully activated states which can have either zero (*R*_0*n*_) or one (*R*_1*n*_) substrate molecule bound (cf. Figure 1A). Substrate molecules are sup-plied at rate *ν*_*i *_and product molecules are used by downstream reactions with specific rate *k_d_*. The inhibition of the PFK by ATP at high ATP concentrations is neglected in the Sel'kov model.

Under steady-state conditions for the enzyme binding reactions the local dynamics of the PFK effectors ATP and ADP is described by the (dimensionless) set of equations [12]

(3) $$\frac{d}{dt}\alpha =v-\sigma \phi_{S},\;\frac{d}{dt}\gamma =q\sigma \phi_{S}-\gamma$$

(4) $$\phi_{S}=\frac{\alpha \gamma^{n}}{1+(1+\alpha )\gamma^{n}}$$

where *ϕ_S _*denotes the fractional saturation. Substrate (*α *= *ATP*/*K_M _*) and product $\left(\gamma =ADP/K_{P}^{app}\right)$ concentrations are measured in terms of the Michaelis-Menten constant $K_{M}=(k+k_{S}^{-})/k_{S}^{+}$ and the apparent dissociation constant for product binding $K_{P}^{app}={\left(k_{P}^{-}/k_{P}^{+}\right)}^{1/n}$. The other parameters are given by $\nu =\nu_{i}/K_{M}k_{d},\;\;\sigma =ke_{0}/K_{M}k_{d}$ and $q=K_{M}/K_{P}^{app}$. Time is measured in units of 1/*k_d _*and *e*_0 _denotes the total enzyme concentration.

#### MWC Model

Based on experimental evidence Goldbeter proposed an alternative approach to describe the allosteric regulation of the PFK [13] which utilizes the Monod-Wyman-Changeux mechanism [37]. Here, the free form of the oligomeric enzyme performs concerted transitions between a catalytically active (*R*_00_) and a catalytically inactive (*T*_00_) conformation where the allosteric constant *L *= *k*^+^/*k^- ^*defines the equilibrium between both conformations in the absence of any ligands (Figure 1B). The enzyme is activated by sequential binding of product molecules with dissociation constant $K_{P}=(k_{P}^{-}/k_{P}^{+})$ for each binding step. Hence, there are *n *+ 1 active enzyme forms *R*_0*m *_to which substrate molecules can bind to form *n *+ 1 enzyme-substrate complexes *R*_1*m*_. Each complex can release product molecules at the specific rate *k*.

Similar as in the Sel'kov model substrate molecules are supplied at rate ν*_i _*and product molecules leave the system with specific rate *k_d _*such that the local dynamics of ATP and ADP is described by the same set of equations as in Eqs. 3 with *ϕ_S _*(Eq. 4) being replaced by (see *Methods*)

(5) $$\phi_{M}=\frac{\alpha {\left(1+\gamma \right)}^{n}}{L+(1+\alpha ){\left(1+\gamma \right)}^{n}}.$$

The parameters have the same meaning as in Eqs. 3 if the apparent dissociation constant $K_{P}^{app}$ is replaced by the true dissociation constant *K_P _*.

Compared with the original Goldbeter model we have neglected the cooperativity with respect to substrate binding and the inhibitory effect of ATP on the PFK activity (as suggested by numerical simulations [32]). With these simplifications we treat the Sel'kov and the Goldbeter model on an equal footing which allows for a direct comparison between their PFK activation mechanisms. Since our model retains the MWC mechanism for PFK activation as an essential part we shall call it the MWC model. We also remark that the PFK actually exhibits sigmoidal behavior with respect to its second substrate fructose-6-phosphate while it does not show any co-operativity with respect to ATP [46,47]. Hence, the simplifying assumption of a hyperbolic dependence of the PFK activity on ATP as a substrate seems to be reasonable.

#### Diffusion and Unified Description

The simplest way to incorporate diffusive coupling between the PFK effectors ATP and ADP is to add 'diffusion terms' in Eqs. 3 with constant (effective) diffusion coefficients. Thereby, we neglect complications arising from allosteric interactions between the PFK effectors and the enzyme which may lead to cross-diffusion terms (non-diagonal elements in the diffusion matrix *D*) and a dependence of the effective diffusion coefficients on the effector concentrations [48,49]. For a recent review of the effects of cross-diffusion on pattern formation see Ref. [50].

Due to the structural similarity between the two expressions in Eqs. 4 and 5 it is convenient to rewrite the effective PFK reaction rate in a unified form. The resulting reaction-diffusion equations read

(6) $$\partial_{t}\alpha =\delta \nabla_{\text{x}}^{2}\alpha +\nu -\sigma \phi_{i}$$

(7) $$\begin{array}{l} \partial_{t}\gamma =\nabla_{\text{x}}^{2}\gamma +q\sigma \phi_{i}-\gamma \\ \phi_{i}=\frac{\alpha {\left(\varepsilon_{i}+\gamma \right)}^{n}}{L_{i}+(1+\alpha ){\left(\varepsilon_{i}+\gamma \right)}^{n}}\text{,}\;\;i=S,\;M \end{array}$$

where the parameter *δ *≡ *D*_ATP_/*D*_ADP _denotes the ratio between the effective diffusion coefficients of inhibitor and activator. Length scales are measured in units of the activator diffusion length given by (*D*_ADP_/*k_d_*)^1/2 ^= (*D*_ATP_/*δk_d_*)^1/2^.

Eqs. 6 and 7 will be analyzed near a supercritical Hopf bifurcation where the dynamics is well de-scribed by the CGLE in Eq. 2. We are particularly interested in the type and the stability of the emerging patterns as we change from a sequential activation mechanism (*L_M _*= *L >*1, *ε_M _*= 1) to a Hill-like activation mechanism (*L_S _*≡ 1, *ε_S _*= 0). Note that the Hill mechanism leads to a factor *γ^n ^*in Eq. 7 while the sequential mechanism produces a factor (1 + *γ*)*^n^*. The latter results from the (binomial) summation over the intermediate enzyme states *R_l0_*, ..., *R_ln _*(*l *= 0, 1) (see *Methods*).

#### Transition from the MWC to the Sel'kov Model

Given the structural similarity between the Sel'kov and the MWC model it will be beneficial to investigate the relation between the two models in more detail. In particular, we expect that the MWC mechanism reduces to that of the Sel'kov model as the affinity for subsequent product binding steps increases (i.e. *K_P _*decreases) such that the product activation becomes more and more cooperative.

To show this explicitly we note that under the rescaling *K_P _*→ *εK_P _*with 0 *< ε <*1 the normalized activator concentration *γ *= *ADP/K_P _*changes as *γ *→*γ*/*ε *and *ϕ_M _*becomes *ϕ_M _*(*α,γ/ε*): = *ϕ_ε _*(*α*,*γ*) with

(8) $$\phi_{\varepsilon }(\alpha ,\gamma )=\frac{\alpha {\left(\varepsilon +\gamma \right)}^{n}}{L_{M}\varepsilon^{n}+(1+\alpha ){\left(\varepsilon +\gamma \right)}^{n}}.$$

Hence, *ϕ_ε _*interpolates between *ϕ_M _*and *ϕ_S _*since *ϕ*_1 ≡ _*ϕ_M _*and as *ε *→ 0 (the binding a finity in-creases) *ϕ_ε _*approaches *ϕ_S _*provided that the product *L_M _ε^n ^*converges to *L_S _*= 1. However, this means that in the MWC model the enzyme cooperativity, as measured by the allosteric constant *L_M _*, has to become increasingly large which is in agreement with the idea that the product activation becomes more cooperative as we change from the MWC to the Sel'kov mechanism.

Formally, we can describe this transition by

(9) $$\underset{\varepsilon \to 0}{\lim}\phi_{\varepsilon }=\phi_{S}\;\text{provided}\;\text{that}\;\underset{\varepsilon \to 0}{\lim}L_{M}\varepsilon^{n}=1.$$

This relation between the MWC and the Sel'kov model will be helpful when we analyze how the type and the stability of the spatio-temporal patterns changes as we change the PFK activation mechanism from the MWC to the Hill type.

## Results

The diffusive coupling of locally oscillatory reactions as in Eqs. 6 and 7 can generate different types of reaction-diffusion wave patterns which can be broadly classified into outward and inward propagating waves [45]. Near a supercritical Hopf bifurcation the transition between these wave types occurs for *c*_1 _-*c*_3 _= 0 (Eq. 2). Depending on the initial and/or boundary conditions these waves may appear in the form of circular or spiral shaped waves.

More complex dynamic behavior can occur near a Benjamin-Feir instability which is indicated by the condition 1 + *c*_1_*c*_3 _*<*0 [42]. In this bifurcation plane wave solutions become unstable against long wave length perturbations which may result in the occurrence of spatio-temporal chaos.

Finally, when the spatial scale separation *δ *becomes sufficiently large the oscillatory instability may be suppressed and stationary Turing patterns can emerge. The transition between wave dynamics and stationary patterns is indicated by the codimension-two Turing-Hopf bifurcation.

To compare the spatio-temporal dynamics of the Sel'kov and the MWC model we have calculated the two CGLE coefficients (*c*_1 _and *c*_3_) and the Turing-Hopf curve for Eqs. 6 and 7 as a function of the systems parameters *L_i_*, *ε_i_*, *ν*, *q*, *n *and *δ *(see Methods). Note that *σ *has been eliminated by the requirement for the system to be near the Hopf bifurcation.

### Sequential vs. Hill-like Activation Mechanism

To decouple the two limiting prescriptions in Eq. 9 we study Eqs. 6 and 7 first in the regime of low glycolytic flux as it is typically observed during oscillatory behavior [51]. This regime is characterized by the conditions *α_s _*= *O*(1) and $\gamma_{s}^{n}\ll 1$ for the Sel'kov and (1 + γ*_s_*)*^n ^*≪ *L *for the MWC model. Here, *α_s _*and *γ_s _*are the respective inhibitor and activator concentrations of the (unstable) steady state. In this approximation, the function *ϕ_i _*in Eq. 7 becomes [12]

(10) $$\phi_{i}\approx \frac{1}{L_{i}}\alpha \;{\left(\in i+\gamma \right)}^{n},\;i=S,\;M.$$

**Figure 2:**
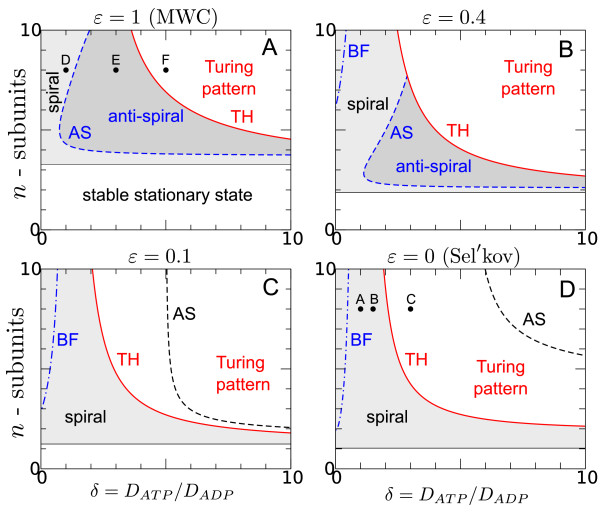
**Transition from a sequential (*ε_M _*= 1) to a Hill-like (*ε_S _*= 0) activation mechanism**. The solid line (Turing-Hopf (TH) bifurcation) separates wave patterns (grey shaded area) from stationary Turing patterns (white area above the TH curve). The dashed line (AS) indicates the transition from inward (dark grey) to outward (light grey) propagating waves where *c*_1 _- *c*_3 _= 0. The dash-dotted line marks the Benjamin-Feir (BF) instability and the occurrence of spatio-temporal chaos nearby. Other parameters: *ν *= 0.5, *q *= 1.

The parameter *L_i _*can be absorbed into the definition of the new parameter combination $\bar{\sigma }=\sigma /L_{i}$ in Eqs. 6 such that we can simply change from the MWC to the Hill mechanism by decreasing *ε *from *ε_M _*= 1 to *ε_S _*= 0 (Figure 2).

As the affinity for the sequential binding of product molecules increases (*ε *decreases) the stability region of inward propagating waves (dark shaded area) decreases (Figure 2A and 2B). At *ε *= 0.1 the transition curve between outward and inward propagating waves (*c*_1 _- *c*_3 _= 0, dashed line) has crossed the Turing-Hopf bifurcation line (Figure 2C). Thus, for *ε *≤ 0.1 the transition to inward propagating waves occurs in the non-oscillatory regime where wave behavior (shaded area) is suppressed in favor of stationary Turing patterns. This shows that the inward propagating waves, as predicted by the CGLE, are not necessarily observable at the level of the original reaction-diffusion system (Eqs. 6 and 10). Since the binding of subsequent product molecules becomes more cooperative as *ε *→ 0 the occurrence of inward propagating waves in the MWC model seems to be related to the sequential activation of the PFK (Figure 1B) which exhibits less cooperativity because the binding a finity (1/*εK_p_*) is finite for *ε *= 1.

### Wave Stability and Numerical Simulations

**Figure 3:**
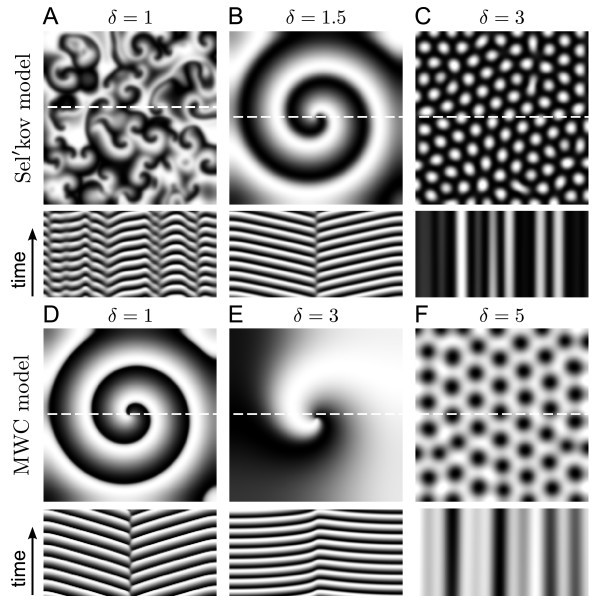
**Numerical simulations of Eqs. 6 and 7.** Spatio-temporal chaos (A), spiral waves (B and D), anti-spiral wave (E) and Turing patterns (C and F) as indicated in the phase diagrams shown in Figure 2A and 2D. Upper panels display snapshots of 2 d simulations. Shown are normalized ATP concentrations (maximal value = white). Lower panels display space-time plots along the dashed line in the respective upper panel (Simulation time: 22 units (A-C), 44 units (D-F)). Note the direction of wave propagation which is outward (away from the spiral core) for B and D, and which is inward in E. Simulations were done on a spatial grid (176 x 176) with no-flux boundary conditions. Parameters: *ν *= 0.5, *q *= 1, *n *= 8 (A-F) and *L *= 10^3 ^(D-F). Parameter *σ *was chosen near the Hopf bifurcation: *σ *= 1770 (A-C), *σ *= 50 (D), = 60 (E and F). Further details are given in *Methods:Numerical Simulations *and Table 1.

For the MWC mechanism outward propagating waves are stable even if the activator diffuses faster than the inhibitor (*δ <*1). However, as *ε *decreases these waves become unstable as indicated by the appearance of a Benjamin-Feir (BF) instability (Figure 2B, 2C and 2D, dash-dotted line). The BF in-stability (1 + *c*_1_*c*_3 _*<*0) marks the region in parameter space where plane waves with a wave number *k *= 0 become unstable to long wave length perturbations. Hence, in the region to the left of the BF curve no stable wave patterns are observable. How-ever, since even for 1 + *c*_1_*c*_3 _*>*0 waves with a finite wave number *k *≠ 0 also can become unstable [41] there is typically a whole region of unstable wave behavior (extending to the right of the BF curve) where spatio-temporal chaos emerges (Figure 3A). Thus, increasing the cooperativity for the product activation steps (*ε *→ 0) leads to a destabilization of the coherent wave behavior for *δ *≤ 1.

**Figure 4:**
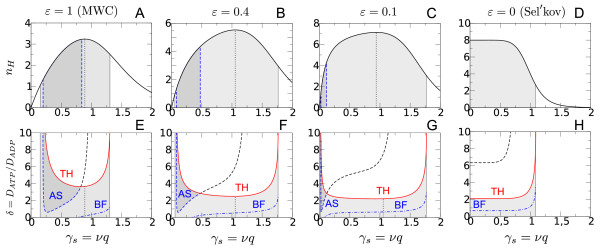
**Transition from the MWC to the Sel'kov mechanism as described by Eq. 9**. Upper panels show the Hill coefficient *n_H _*(Eq. 11) as a function of the steady state activator concentration *γ_s _*while the lower panels show the respective phase diagram. The solid line (TH) separates wave patterns (grey shaded area) from stationary Turing patterns. The dashed line indicates the transition from inward (dark grey) to outward (light grey) propagating waves while the dash-dotted line marks the Benjamin-Feir (BF) instability and the occurrence of spatio-temporal chaos. The dotted line marks the location of the maximum of the Hill coefficient. Other parameter: *L *= 10^3^, *n *= 8, *q *= 1.

Away from the BF instability curve (*δ >*1) outward propagating waves become stable even in the limit *ε *= 0 (Figure 3B). As *δ *increases further these waves turn into stationary Turing patterns (Figure 3C). In contrast, for the sequential product activation mechanism (*ε *= 1) there is no Benjamin-Feir instability for typical numbers of PFK subunits *n *≤ 8 [52] and sufficiently low values of the steady state activator concentration *γ_s _*= *νq *(cf. Figure 4E). Hence, there is no spatio-temporal chaos in that regime. Instead, outward propagating waves are stable at low values of *δ *(Figure 2A and Figure 3D). As *δ *increases the direction of wave propagation changes from outward to inward such that anti-spirals become observable (Figure 3E). At sufficiently large values of *δ *wave behavior is suppressed by a Turing instability (Figure 3F) similar as for the Sel'kov model.

### Inward Propagating Waves and Enzyme Cooperatively

The fact that the occurrence of inward propagating waves depends on the binding a finity for sub-sequent product activation steps suggests that the strength of enzyme cooperativity might play a role in this respect. The amount of cooperativity can be conveniently quantified by an effective Hill coefficient which is defined (with respect to the activator concentration) as [40]*n_H _*= (*γ/M*)(*dM/dγ *) where *M *≡ *ϕ*/(1 - *ϕ*). For *ϕ *= *ϕ_i _*(Eq. 7) this quantity is explicitly given by

(11) $$n_{H}(\gamma )=\frac{\gamma }{\varepsilon_{i}+\gamma }\frac{nL_{i}}{L_{i}+{\left(\varepsilon_{i}+\gamma \right)}^{n}},\;\;\;i=S,M.$$

Positive (negative) cooperativity corresponds to values *n_H _>*1 (*n_H _<*1) while *n_H _*= 1 indicates no cooperativity.

To perform the transition from the MWC to the Hill mechanism when *L *is not necessarily large we introduce in Eq. 8 an effective allosteric constant as *L_eff _*≡ *L_M _ε^n ^*= *ε *(*L - *1) + 1. This definition ensures that *L_eff _*has the correct limiting behavior as required by Eq. 9, i.e. *L_eff _*= *L_M _*= *L *for *ε *= 1 and *L_eff _*→ *L_S _*= 1 as *ε *→ 0. Note that the true allosteric constant *L_M _*increases as 1/*ε^n ^*when *ε *→ 0.

**Figure 5:**
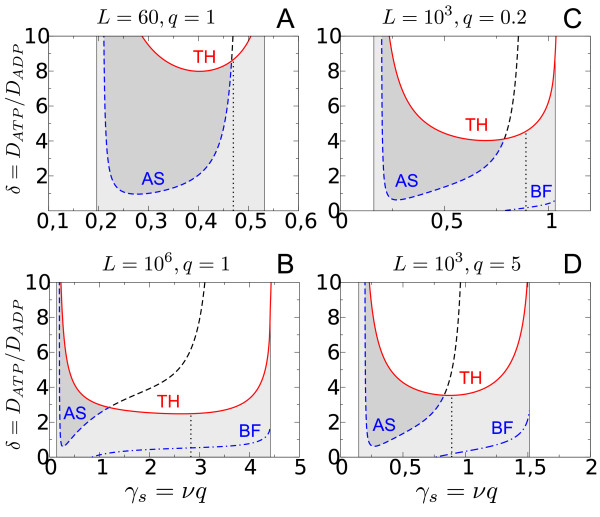
**Phase diagrams similar as in Figure 4E, but for different values of *L *and *q***. The phase diagrams for the occurrence of spiral waves (light grey), anti-spirals (dark grey) and Turing patterns (white area above the TH curve) remain qualitatively the same independent of the particular values of the parameters *L *(A and B) and *q *(C and D). For comparison, see the case *L *= 10^3 ^and *q *= 1 shown in Figure 4E.

In Figure 4 we relate the occurrence of inward propagating waves to the properties of the effective Hill coefficient *n_H _*as described by Eq. 11. For the MWC model *n_H _*exhibits a single maximum as a function of the steady state activator concentration *γ_s _*= *νq *(Figure 4A). While oscillatory behavior (grey shaded area) is observed on both sides of the maximum (dotted line) of the Hill curve inward propagating waves only occur to the left of it where *dn_H_/dγ_s _>*0 (Figure 4E). This behavior is independent of the particular choice of the other parameters *L *and *q *(Figure 5). As *ε *decreases the cooperativity of product activation increases as indicated by an in-crease in the maximum of the Hill curve (Figure 4B and 4C). Concomitantly, the stability region of anti-spiral waves rapidly shrinks and subsequently shifts to small activator concentrations where the steep-ness of the Hill curve is sufficiently large (Figure 4F and 4G). This suggests that it is not the strength of the cooperativity per se, but the sensitivity of the Hill coefficient with respect to changes in the activator concentration which determines whether inward propagating waves can occur or not. This is in agreement with the fact that for the Sel'kov model, where $n_{H}=n/(1+\gamma_{s}^{n})$ is a monotonically decreasing function (*dn_H_/dγ_s _*≤ 0), the formation of inward propagating waves is suppressed (Figure 4D and 4H).

## Discussion

Beginning in the 1960s glycolytic oscillations have become one of the best studied biochemical oscillators both in cell-free extracts [26,38,53] and in living cells [28,54]. Later it was found in studies with yeast cell populations that glycolytic oscillations represent a collective phenomenon. The oscillations in individual cells are synchronized through the exchange of metabolic intermediates such as acetaldehyde [55] or glucose [56]. At low cell densities the oscillations at the population level disappear (synchronously in all cells) indicating a quorum sensing mechanism [57]. Synchronized behavior was also observed in cell-free extracts where diffusive coupling of glycolytic enzymes can generate waves of glycolytic activity [30,31]. However, clear experimental evidence for metabolic waves in living cells remains scarce [58] although mathematical modeling supports the feasability of such waves [59].

Recently, a novel type of wave dynamics, called inward rotating spiral waves, has been observed in cell-free yeast extracts [32]. Such wave behavior has, so far, only been observed in purely chemical systems [35,36]. Here, we have investigated the molecular mechanism underlying the generation of such anti-spiral waves in simple glycolytic model systems which focus on the allosteric activation of the glycolytic enzyme phosphofructokinase (PFK). We have shown that in the Goldbeter model inward rotating spiral waves can arise due to the sequential activation of the PFK implied in the Monod-Wyman-Changeux mechanism [13,37]. In the limit of an in finitely large binding a finity where the PFK activation is described by a Hill function, as in Sel'kov model [12], the capability to generate inward propagating waves is lost. This suggests that the MWC mechanism, as in Figure 1B, can not be further implied. On the other hand, as we have shown earlier [32] the capability to generate anti-waves is retained by the Goldbeter model where the cooperativity with respect to substrate binding and the allosteric inhibition by ATP are additionally taken into account. Hence, the MWC model can be regarded as a 'core' mechanism for the generation of inward propagating waves for allosteric enzyme systems with product activation.

For well-mixed reaction systems a simple Hill function is often employed to model cooperative behavior in a 'generic' way. Near the onset of oscillations choosing a Hill kinetics instead of a more complex activating function, as in the MWC model, does not lead to a qualitative change in the dynamics under well-stirred conditions. However, as we have shown, the choice of the activating function can significantly change the type and the stability of dynamic patterns in the presence of diffusive coupling. For example, the appearance of a Benjamin-Feir instability in the Sel'kov model indicates the occurrence of spatio-temporal chaos which is mostly absent in the MWC model (Figure 2, 3 and 4). This suggests that the intermediate enzyme forms in the MWC model, which are only partially saturated with product molecules, can stabilize the system dynamics against long wave length perturbations.

Sel'kov and Goldbeter have shown that for the PFK mediated reaction to become oscillatory a sufficiently strong positive enzyme cooperativity is required [12,60]. However, as far as oscillations are concerned the detailed shape of the Hill coefficient curve (Eq. 11) is not important. Consequently, they occur on the ascending branch of the cooperativity curve (where *dn_H_/dγ_s _>*0) as well as on the descending branch (where *dn_H_*/*dγ_s _<*0) as long as *n_H _>*1 (Figure 4A, B, C and 4D). Interestingly, the occurrence of inward propagating waves does not seem to depend on the magnitude of the enzyme cooperativity, but on its sensitivity with respect to changes in the activator concentration. Our simulations show that the formation of inward propagating waves correlates with a positive sensitivity (*dn_H_*/*dγ_s _>*0) which indicates that for the pattern forming aspects of allosteric enzyme systems more subtle enzyme properties play a role than they do for the occurrence of oscillations.

Since the glycolytic model systems in Eqs. 6 and 7 are of the substrate-depletion type [14] it is not surprising that both models predict the occurrence of stationary Turing patterns if the spatial scale separation between inhibitor and activator dynamics becomes sufficiently large [49]. What is surprising is the fact that this transition already occurs for comparably small values of *δ *= 2, ..., 4 if the number *n *of enzyme subunits is sufficiently large (Figure 2A and 2D). This strong dependence on the enzyme cooperativity has been largely neglected in earlier work [61,62] which mostly focused on the case *n *= 2 (corresponding to muscle PFK). However, in yeast the PFK is an octamer (*n *= 8) for which Turing pat-terns are predicted to occur for *δ >*4 in the MWC model and for *δ >*2 in the Sel'kov model. The necessary spatial scale separation could be generated, for example, through preferential allosteric interactions of the PFK effectors with immobilized enzymes [32,63], including the PFK itself. Although Turing patterns can be systematically generated only in chemical systems yet [25] our results suggest that high oligomeric enzyme systems are promising candidates to generate such patterns also in properly designed biochemical reaction-diffusion systems.

## Conclusions

In well-mixed reaction systems the systematic investigation of molecular reaction mechanisms has led to considerable insights into the design principles for the generation of a specific type of dynamic behavior such as bistability or oscillations [14,15,64]. Here, we have expanded this approach to the case of spatially extended systems. Specifically, we have demonstrated that amplitude equations are a valuable tool to investigate how the occurrence of particular spatio-temporal patterns depends on the details of the underlying molecular reaction mechanism in the presence of diffusive coupling. In that way we could provide a molecular explanation for the occurrence of inward rotating spiral waves as they were recently observed in glycolysis in cell-free yeast extracts. Our results support the view that in yeast the allosteric enzyme phosphofructokinase is activated by a Monod-Wyman-Changeux and not by a Hill mechanism. They also highlight the importance of the number of enzyme subunits for a possible experimental generation of Turing patterns in biological systems.

## Methods

### Derivation of the rate law for the PFK in the MWC model

In general, if all enzyme binding reactions are in quasi-steady state the effective reaction rate can be written as [65]*v*= *ke*_0_*ϕ*. Here *k *is the intrinsic substrate conversion rate of a single enzyme subunit, *e*_0 _denotes the total enzyme concentration and

(12) $$\phi =\frac{\sum_{m=0}^{n}1\cdot R_{1m}}{T_{00}+\sum_{m=0}^{n}(R_{0m}+R_{1m})}$$

is the fractional saturation function which measures the number of occupied substrate binding sites relative to the total number of substrate binding sites. In quasi-steady state the active enzyme states *R_lm _*can expressed in terms of binding constants and substrate/product concentrations. For example, for the case *n *= 2 shown in Figure 1B we have *R*_01 _= 2*R*_00_*ADP/K_S _*and *R*_02 _= *R*_01_*ADP/*2*K_S _*= *R*_00 _(*ADP/K_S_*)^2^. Hence, the summation over the intermediate enzyme states in $\sum_{l=0}^{2}R_{0l}={\left(1+ADP/K_{S}\right)}^{2}\;\;R_{00}$ produces a binomial series. Similarly, we obtain $\sum_{l=0}^{2}R_{1l}={\left(1+ADP/K_{S}\right)}^{2}\;R_{00}(ATP/K_{M})\;$ such that *ϕ *as defined in Eq. 12 reproduces Eq. 5 by taking into account that *L *= *T*_00_/*R*_00_.

### Calculation of the Hopf and the Turing Instability

We begin by rewriting Eqs. 6 and 7 in the form

(13) $$\begin{array}{l} \partial_{t}\alpha \;=\;\delta \nabla_{x}^{2}\alpha +f_{\alpha }(\alpha ,\gamma ;p)\\ \partial_{t}\gamma \;=\;\nabla_{x}^{2}\gamma +f_{\gamma }(\alpha ,\gamma ;p) \end{array}$$

where the functions *f_α _*and *f*_γ _are given by

$$\begin{array}{l} f_{\alpha }(\alpha ,\gamma ;p)\;=\;\nu -\sigma \phi_{i}(\alpha ,\gamma )\\ f_{\gamma }(\alpha ,\gamma ;p)\;=\;q\sigma \phi_{i}(\alpha ,\gamma )-\gamma . \end{array}$$

The function *ϕ_i _*is defined as

$$\phi_{i}(\alpha ,\gamma )=\frac{\alpha {\left(\varepsilon_{i}+\gamma \right)}^{n}}{L_{i}+(1+\alpha ){\left(\varepsilon_{i}+\gamma \right)}^{n}}\;\;\;i=S,\;M,$$

where the Sel'kov model is characterized by *ε_S _*= 0 and *L_S _*= 1 while the Monod-Wyman-Changeux model is obtained for *ε_M _*= 1 and *L_M _*= *L >*1. In Eqs. 13 the vector *p *= (ν, *σ *, *q*, *n*, *L_i_*, *ε_i_*) collectively denotes the kinetic parameters appearing in the functions *f*_α _and *fγ*.

#### Steady States

The unique (spatially homogeneous) steady state of Eqs. 13 is given by

(14) $$\begin{array}{cc} \alpha_{s}=\frac{v}{\sigma -v}\left(1+\frac{L_{i}}{{\left(\varepsilon_{i}+vq\right)}^{n}}\right), & \gamma =vq \end{array}.$$

For *α_s _>*0 to be positive we require that *σ > ν*.

#### Turing and Hopf bifurcation thresholds

The stability of the fixed point (*α_s_*, *γ_s_*) against spatio-temporal perturbations of the form *δ*α (*x*, *t*) =*α*_0 _exp (*ikx *+ λ*t*) and *δγ*(*x*, *t*) = *γ*_0 _exp (*ikx *+ λ*t*) is determined by the characteristic polynomial

(15) $$\lambda^{2}+a_{1}(k)\lambda +a_{0}(k)=0$$

where *λ*(*k*) characterizes the temporal evolution of a spatial growth mode with wave vector *k*. The co-efficient functions *a*_0 _and *a*_1 _in Eq. 15 are given by

$$\begin{array}{l} a_{1}(k)\;=\;k^{2}(1+\delta )+1-qB+A\\ a_{0}(k)\;=\;\delta k^{4}+k^{2}(\delta (1-qB)+A)+A. \end{array}$$

Where

(16) $$\begin{array}{l} A\;=\;\frac{{\left(\sigma -\nu \right)}^{2}}{\sigma (1+L_{\text{eff}})}\text{,}\;\;\;L_{\text{eff}}\equiv \frac{L_{i}}{{\left(\varepsilon_{i}+\nu q\right)}^{n}}\\ B\;=\;\frac{(\sigma -\nu )\nu }{\sigma }\;\frac{nL_{\text{eff}}}{\left(\varepsilon_{i}+\nu q)(1+L_{\text{eff}}\right)}. \end{array}$$

Instabilities occur if there exists a *k_c _*for which *Re *(λ(*k_c_*)) *>*0. The type of instability depends on whether this occurs for *k_c _*= 0 (Hopf bifurcation) or for *k_c _*≠ 0 (corresponding to a Turing bifurcation if, in addition, *Im *(λ(*k_c_*)) = 0).

##### Turing bifurcation

The critical wave number *k_c _*of the most unstable mode in the Turing bifurcation is determined by *da*_0_/*dk *= 0, and the corresponding parameter set is implicitly given by *a*_0_(*k_c_*) = 0.

Hence,

$$k_{c}^{2}=\frac{1}{2\delta }[\delta (qB-1)-A]$$

and the parameter set for the Turing bifurcation is described by

(17) $$T(\delta ,p)={\left[\delta (qB-1)-A\right]}^{2}-4\delta A=0.$$

##### Hopf bifurcation

The Hopf bifurcation is determined by *a*_1_(*k *= 0) = 0 or 1 + *A - qB *= 0 which is a quadratic equation for *σ*. The respective solution is given by

(18) $$\sigma_{H}=r-\sqrt{r^{2}-s}$$

With

$$\begin{array}{l} r\;=\;\nu +\frac{1}{2}\left(L_{\text{eff}}\left[\frac{(n-1)\nu q-\epsilon_{i}}{\epsilon_{i}+\nu q}\right]-1\right)\\ s\;=\;\nu^{2}\left(1+L_{\text{eff}}\frac{nq}{\varepsilon_{i}+\nu q}\right). \end{array}$$

##### Turing-Hopf codimension-2 bifurcation

In general, oscillations are observed for *σ *≤ *σ_H _*if *σ_H _< σ_T _*while Turing patterns emerge for *σ *≤ *σ_T _*if *σ_T _< σ_H _*and *σ_T _*is the smallest (real) root of Eq. 17. However, if both of these codimension-1 bifurcations occur simultaneously (*σ_H _*= *σ_T _*) a Turing-Hopf codimension two bifurcation takes place. An implicit expression for this bifurcation curve is obtained by using the explicit representation for *σ_H _*(Eq. 18) in the expression for *T *(*δ*, *p*) = 0 (Eq. 17). Note that near the Turing-Hopf bifurcation curve it can be difficult to predict whether wave or Turing patterns are observed since both can be simultaneously stable. Alternatively, mixed mode patterns can appear near a Turing-Hopf bifurcation [66].

### Calculation of the CGLE coefficients *c*_1 _and *c*_3_

Near the supercritical Hopf bifurcation the spatio-temporal dynamics of the reaction-diffusion system in Eqs. 13 is well described by the complex Ginzburg Landau equation (CGLE) [41]

$$\partial_{t}A=(1+ic_{1})\nabla_{x}^{2}A+A-(1+ic_{3})|A{|}^{2}A.$$

Here, *A*(*x*, *t*) is a complex amplitude describing slow spatio-temporal modulations around the (spatially homogeneous) unstable steady state of Eqs. 13 while *c*_1 _and *c*_3 _are real coefficients. In general, *c*_3 _= *c*_3_(*p*) only depends on the reaction mechanism through the kinetic parameters while *c*_1 _= *c*_1_(*p*,*δ *) additionally depends on the ratio of the diffusion coefficients *δ *= *D*_α_/*D_γ_*.

To determine the borderline between inward and outward propagating waves, given by *c*_1 _- *c*_3 _= 0, we will calculate the two CGLE coefficients *c*_1 _and *c*_3 _as a function of the original system parameters following the approach in Ref. [41].

#### Calculation of c_1_

The first CGLE coefficient *c*_1 _is given by *c*_1 _= *d*_2_/*d*_1 _where $d=d_{1}+id_{2}=u_{0}^{*}Du_{0}$. Here, *i *is the imaginary unit, *D *= *diag*(*δ*, 1) denotes the diagonal diffusion matrix and $u_{0}^{*}(u_{0})$ are the left(right) eigenvectors of the Jacobian matrix

$$L_{0}=\left(\begin{array}{cc} f_{\alpha ,\alpha } & f_{\alpha ,\gamma }\\ f_{\gamma ,\alpha } & f_{\gamma ,\gamma } \end{array}\right)=\left(\begin{array}{cc} -A & -\frac{1+A}{q}\\ qA & A \end{array}\right)$$

where *f*_α_,_α _≡ ∂*f*_α_/∂*α*, etc. denote the respective partial derivate. Here and in the following all expressions have to be evaluated at the Hopf bifurcation by eliminating from the expression for *A *using Eqs. 16 and 18. The eigenvectors

$$\begin{array}{l} u_{0}=\left(\begin{array}{c} \frac{1}{q}\left(-1+\frac{i}{\sqrt{A}}\right)\\ 1 \end{array}\right)\\ u_{0}^{*}=\frac{1}{2}\left(-iq\sqrt{A},1-i\sqrt{A}\right) \end{array}$$

are normalized as $u_{0}^{*}\cdot \;u_{0}=\text{ 1}$ and their respective eigenvalues are given by $\lambda_{0}=i\sqrt{A}$ and $\lambda_{0}^{*}=-i\sqrt{A}$. Hence, the oscillation frequency at the Hopf bifurcaption is given by $\omega_{H}=\sqrt{A}$. For *c*_1 _we find

(19) $$c_{1}=\omega_{H}\frac{\delta -1}{\delta +1}.$$

#### Calculation of c_3_

The calculation of *c*_3 _is more tedious, but straight-forward [41]. The expression for *c*_3 _is given by *c*_3 _= *g*_2_/*g*_1 _where

(20) $$\begin{array}{c} g_{1}+ig_{2}=-2u_{0}^{*}M_{0}u_{0}V_{0}-2u_{0}^{*}M_{0}{\bar{u}}_{0}V_{+}\\ -3u_{0}^{*}N_{0}u_{0}u_{0}{\bar{u}}_{0}. \end{array}$$

The 'overbar' in $\bar{u_{0}}$ denotes complex conjugation and transposition such that $\bar{u_{0}}$ is a column vector. In Eq. 20 we have defined the tensor-valued vectors *M*_0 _= (*M*_1_, *M*_2_) and *N*_0 _= (*N*_1_, *N*_2_). The components of *M*_0 _are given by the second-rank tensor

$$\begin{array}{l} M_{1,ij}=\frac{1}{2}\frac{\partial^{2}f_{\alpha }}{\partial x_{i}\partial x_{j}}{|}_{\left(\alpha_{\text{s}},\gamma_{\text{s}},\sigma_{H}\right)}\\ \;\;\;\;\;\;\;\;\;\;\;=\;-\frac{\sigma_{H}}{2}\frac{\partial^{2}\phi_{i}}{\partial x_{i}\partial x_{j}}{|}_{\left(\alpha_{\text{s}},\gamma_{\text{s}},\sigma_{H}\right)}=-\frac{M_{2,ij}}{q} \end{array}$$

while each component of *N*_0 _is a third-rank tensor given by

$$\begin{array}{l} N_{1,ijk}=\frac{1}{6}\frac{\partial^{3}f_{\alpha }}{\partial x_{i}\partial x_{j}\partial x_{k}}{|}_{\left(\alpha_{\text{s}},\gamma_{\text{s}},\sigma_{H}\right)}\\ \;\;\;\;\;\;\;\;\;\;\;\;=\;-\frac{\sigma_{H}}{6}\frac{\partial^{3}\phi_{i}}{\partial x_{i}\partial x_{j}\partial x_{k}}{|}_{\left(\alpha_{\text{s}},\gamma_{\text{s}},\sigma_{H}\right)}=-\frac{N_{2,ijk}}{q}. \end{array}$$

Here we have set (α, γ) ≡ (*x*_1_, *x*_2_) and *i, j, k *= 1, 2. Hence, in component form Eq. 20 reads

$$\begin{array}{l} g_{1}+ig_{2}=-2u_{0l}^{*}M_{0l,ij}u_{0i}V_{0j}-2u_{0l}^{*}M_{0l,ij}{\bar{u}}_{0i}V_{+j}\\ \;\;\;\;\;\;\;\;\;\;\;\;\;\;\;\;\;\;\;\;-3u_{0l}^{*}N_{0l,ijk}u_{0i}u_{0j}{\bar{u}}_{0k} \end{array}$$

where we have used the Einstein summation convention according to which over indices appearing twice in a term has to be summed automatically - from 1 to 2 in our case. The vectors *V*_0 _and *V*_+ _are given by

$$V_{0}\;=\;-2L_{0}^{-1}M_{0}u_{0}{\bar{u}}_{0}$$

$$V+\;=\;-{\left(L_{0}-2\lambda_{0}Id\right)}^{-1}M_{0}u_{0}u_{0}$$

where *Id *≡ *diag*(1, 1) denotes the 2 × 2 identity matrix. The matrix *L*_0 _and the eigenvalue *λ*_0 _have been defined in Section.

##### Explicit expression for *c*_1 _and *c*_3 _in the limit of low glycolytic flux

The calculation of *c*_3 _has been automatized using a computer algebra system which was also used to plot the graphs in Figure 2 and 4. In contrast to *c*_1 _(Eq. 19) the output for *c*_3 _is too clumsy to be displayed in a comprehensive manner. How-ever, in the case of low glycolytic flux where Eqs. 13 can be approximated as (cf. Eq. 10)

$$\begin{array}{l} \partial_{t}\alpha =\delta \nabla_{\text{x}}^{2}\alpha +\nu -\bar{\sigma }\alpha {\left(\varepsilon_{i}+\gamma \right)}^{n},\;\;\;\;\bar{\sigma }=\sigma /L\\ \partial_{t}\gamma =\nabla_{\text{x}}^{2}\gamma +q\bar{\sigma }\alpha {\left(\varepsilon_{i}+\gamma \right)}^{n}-\gamma \end{array}$$

the expression for *c*_3 _considerably simplifies to

$$c_{3}=\frac{P_{2}(n){\left(\nu q\right)}^{2}+\varepsilon_{i}P_{1}(n)\nu q+\varepsilon_{i}^{2}P_{0}(n)}{P_{3}(n,\nu q,\varepsilon_{i})(\nu q+\varepsilon_{i})\omega_{H}}$$

where the polynomial functions *P_i _*are given by

$$P_{0}=\text{ 9}n\;–\text{3}$$

$$P_{\text{1}}=\;–\text{ 9}n^{\text{2}}+\text{ 13}n\;–\text{6}$$

$$P_{\text{2}}=\text{ 2}n^{\text{3}}–\text{5}n^{\text{2}}+\text{ 6}n\;–\text{3}$$

$$P_{\text{3}}=\text{ 3 }{\left(n\;–\text{1}\right)}^{\text{2}}(nq)\;–\text{ 3}\varepsilon_{i}(n\;–\text{3}).$$

The expression for *c*_1 _is still given by Eq. 19 with the Hopf frequency

$$\omega_{H}^{2}=\frac{\nu q(n-1)-\varepsilon_{i}}{\nu q+\varepsilon_{i}}.$$

Note, that for the Sel'kov case, where *ε_i _*≡ *ε_S _*= 0, the coefficients *c*_1 _and *c*_3 _become independent of the parameter combination *νq *corresponding to the steady state value of the activator concentration.

### Numerical Simulations

The results of numerical simulations shown in Figure 3 were generated by discretizing Eqs. 6 and 7 in space and time where the Laplacian was approximated by finite differences (five-point-scheme). The resulting set of ordinary differential equations was integrated using a 4th order Runge-Kutta scheme with no-flux boundary conditions. The respective parameters are summarized in Table 1. The Turing patterns in Figure 3C and 3F were generated from random initial conditions chosen as

**Table 1:** Parameters for the Numerical Simulations shown in Figure 3.

model	Sel'kov			MWC		
**panel**	**A**	**B**	**C**	**D**	**E**	**F**

system parameters						

*δ*	1	1.5	3	1	3	5

*σ*	1770	1770	1770	50	60	60

*α_s _*(Eq. 14)	0.073	0.073	0.073	0.4	0.34	0.34

*γ_s _*(Eq. 14)	0.5	0.5	0.5	0.5	0.5	0.5

integrator parameters						

spatial step: *dx *= *dy*	3	3	0.3	3	10	0.3

time step: *dt*	0.05	0.05	3.10^-3^	0.05	0.1	3.10^-3^

side length *l_s_*	528	431	30.5	528	10^3^	23.6

For the integration of Eqs. 6 and 7 we have fixed the following parameters *ν *= 0.5, *q *= 1, *L*_*S *_= 1, *L*_*M *_= 10^3^, *ε_S _*= 0, *ε_M _*= 1 and *n *= 8 (number of PFK subunits). Simulations were done on a square grid of dimension *N *× *N *with *N *= 176. The dimensionless side length of the domain is given by *l_s _*= *dx · N/δ*^1/2^.

$$\alpha (t=0,x,y)\;=\alpha_{s}\;(1+0.05(2r-1)),$$

where *r *is a random number equally distributed in the interval [0,1] and *x*, *y *= 1, ...,*N*. A similar expression was used for *γ*.

To generate a (anti-)spiral wave as in Figure 3A, D and 3E one has note that the spiral core corresponds to a phase singularity [41]. If not created initially such a phase defect will not develop spontaneously in a spatially homogeneous medium. To simulate the spiral waves we have, thus, created a phase defect by initially imposing a spatial gradient of the inhibitor (*α*) along the *x*-direction and a second spatial gradient of the activator (*γ*) along the *y*-direction as

$$\begin{array}{l} \alpha (t=0,x,y)\;=\;\alpha_{s}\left(1+\kappa \left(\frac{2x}{N}-1\right)\right)\\ \gamma (t=0,x,y)\;=\;\gamma_{s}\left(1+\kappa \left(\frac{2y}{N}-1\right)\right). \end{array}$$

To generate Figure 3A and 3B we have chosen *k *= 1/70 while Figure 3D and 3E were generated with *k*= 2/3.

We remark that the physical side length of the simulation domain is given by λ*_D _*· *dx *· *N *where

(21) $$\lambda_{D}=\sqrt{\frac{D_{ADP}}{k_{d}}}=\sqrt{\frac{D_{ATP}}{\delta \cdot k_{d}}}$$

denotes the diffusion length of the activator, *dx *is the spatial step size of the discretization and *N *= 176 is the number of grid points which we kept fixed for all simulations. As a result, the physical dimensions of each panel in Figure 3 are different since the simulations were done for different values of the spatial scale separation *δ *= *D_ATP_*/*D_ADP _*and *dx *(cf. Table 1). Once specific values for the ATP diffusion co-efficient *D_ATP _*and the product consumption rate *k_d _*are provided the physical side length is determined by (*D_ATP_*/*k_d_*)^1/2 ^*l_s _*where *l_s _*= *dx *· *N/δ*^1/2 ^denotes the dimensionless side length.

## Authors' contributions

RS conceived the study, performed the analytical calculations, the numerical simulations and drafted the manuscript. EMN helped to interpret the results and contributed to the manuscript. All authors read and approved the final manuscript.
